# Supplementary material for: The Paeonia qiui R2R3-MYB Transcription Factor PqMYBF1 Positively Regulates Flavonol Accumulation
Source: Plants (Basel). 2023 Mar 23;12(7):1427. doi: 10.3390/plants12071427 (PMC10096829; doi:10.3390/plants12071427)
Supplement: Supplementary file 1 [file plants-12-01427-s001.zip › Figures S1-S2.pdf]

```
                10        20        30        40        50        60        70        80        90       100       110       120
1       ATGGGGAGGGCACCCTGTTGTGAGAAAGTGGGATTGAAGAAAGGAAGGTGGACGGCTGAAGAAGATGATATTTTGATCAAGTACATCCAATCTAATGGTGAAGGTTCATGGAGGTCTTTA
1        M  G  R  A  P  C  C  E  K  V  G  L  K  K  G  R  W  T  A  E  E  D  D  I  L  I  K  Y  I  Q  S  N  G  E  G  S  W  R  S  L

               130       140       150       160       170       180       190       200       210       220       230       240
121     CCTAAAAATGCAGGGCTTTTGAGGTGTGGCAAGAGTTGCAGGCTGAGATGGATAAACTATTTGCGAGGTGACTTGAAGAGGGGGAACATTTCATCTGAAGAAGAAGATATCATCATAAAG
41       P  K  N  A  G  L  L  R  C  G  K  S  C  R  L  R  W  I  N  Y  L  R  G  D  L  K  R  G  N  I  S  S  E  E  E  D  I  I  I  K

               250       260       270       280       290       300       310       320       330       340       350       360
241     CTGCATGCGTCGATTGGAAATAAGTGGTCCTTGATTGCTGGCCAGTTACCAGGAAGGACAGACAACGAAATAAAGAACTACTGGAACAGTCACTTGAGTAGAAAAATCGACACATTTAGA
81       L  H  A  S  I  G  N  K  W  S  L  I  A  G  Q  L  P  G  R  T  D  N  E  I  K  N  Y  W  N  S  H  L  S  R  K  I  D  T  F  R

               370       380       390       400       410       420       430       440       450       460       470       480
361     AGGCCAATAAGCCAGACTCCACCCATTGTTTTTAACTTAGCCAAGATGGACTCTGTTCCGTCCAAGAGAAAAGGAGGCAGGACAAGCAGAGCAGCCATGAAGAAGAACAAAACCTACAAC
121      R  P  I  S  Q  T  P  P  I  V  F  N  L  A  K  M  D  S  V  P  S  K  R  K  G  G  R  T  S  R  A  A  M  K  K  N  K  T  Y  N

               490       500       510       520       530       540       550       560       570       580       590       600
481     CGCCTCACACCAAAAGTCAAGGAAAAGGACAATTGTGTTGACTCAGTCAAAGTGACCATTCCATGGCAGCCAGAGCAAGACGACATGGTTTTGGATACGTGGCCTCAAGGTGGTGGAATG
161      R  L  T  P  K  V  K  E  K  D  N  C  V  D  S  V  K  V  T  I  P  W  Q  P  E  Q  D  D  M  V  L  D  T  W  P  Q  G  G  G  M

               610       620       630       640       650       660       670       680       690       700       710       720
601     TTGGATCCAAGTGAGGAGGAGAGAGGAAGTTGTGAGGAGAGAGAGAGCAGGAATTCAATGCCGTGCCCTGTTGTTGAAAGAGAGGGTGAAATGTTGGGACCGTGTGAAGAGCTGGAAAAT
201      L  D  P  S  E  E  E  R  G  S  C  E  E  R  E  S  R  N  S  M  P  C  P  V  V  E  R  E  G  E  M  L  G  P  C  E  E  L  E  N

               730       740       750       760       770       780       790       800       810       820       830       840
721     GGGATGTTGTCTTTTAATGACATTATAGGAAATGAATTGCTTCTGGATGCAAATGGCAATTTGACGGTGACTGGTAATGATGACCAAAAAAATTACTTAGTTACTTCGGATTCAAATTCA
241      G  M  L  S  F  N  D  I  I  G  N  E  L  L  L  D  A  N  G  N  L  T  V  T  G  N  D  D  Q  K  N  Y  L  V  T  S  D  S  N  S

               850       860       870       880       890       900       910       920       930       940       950       960
841     ACCAAAATGGACGATCAAATGGAGGTGAGTGGTAATTTGAGCTCGAATGGAGAGAGTGGTAATAATGCAGAGTGGCTCCCCTGCTCTTCCTCGCATTCGCCGATTTCATATTTTGATGAT
281      T  K  M  D  D  Q  M  E  V  S  G  N  L  S  S  N  G  E  S  G  N  N  A  E  W  L  P  C  S  S  S  H  S  P  I  S  Y  F  D  D

               970       980       990      1000      1010      1020      1030      1040      1050      1060      1070      1080
961     GGAAGGATTGATTGGGATTTGGAAGGGAGTGTCTTGGGGCAAGGGATTTGGGAGGAACAAGAAGATCAGGTTCTGTCATGGTTATGGGACTGTGACAAGGGGGGAGATGACCTTCAAAAT
321      G  R  I  D  W  D  L  E  G  S  V  L  G  Q  G  I  W  E  E  Q  E  D  Q  V  L  S  W  L  W  D  C  D  K  G  G  D  D  L  Q  N

              1090      1100      1110      1120      1130      1140
1081    TTGGGGGGAGAGATGGATTGTGAACGACAAAAAGCTATGGTTGCTTGGCTTCTTTCTTGA
361      L  G  G  E  M  D  C  E  R  Q  K  A  M  V  A  W  L  L  S  *
```

Figure S1. The sequence information of *PqMYBF1*.

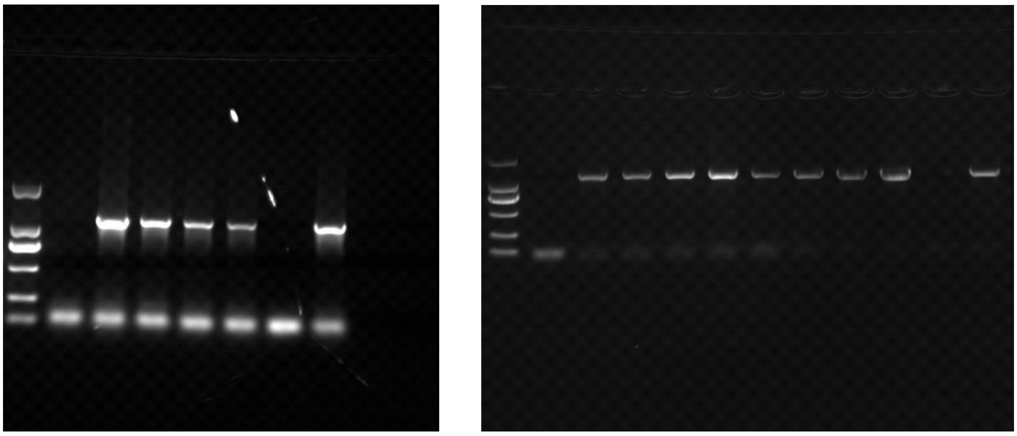

Figure S2. Original Images of gel for transgenic plant identification.
