# Supplementary material for: The Paeonia qiui R2R3-MYB Transcription Factor PqMYBF1 Positively Regulates Flavonol Accumulation
Source: Plants (Basel). 2023 Mar 23;12(7):1427. doi: 10.3390/plants12071427 (PMC10096829; doi:10.3390/plants12071427)
Supplement: Supplementary file 1 [file plants-12-01427-s001.zip › Tables S1-S6.pdf]

Table S1 The primers used for PqMYBF1 gene cloning.

Primer name	Primer sequence (5′-3′)
PqMYBF1- F	GGATCCATGGGGAGGGCACCCTGTTGTGA
PqMYBF1- R	GTCGACTCAAGAAAGAAGCCAAGCAACCA

Table S2 The gene specific primers used for qRT-PCR analysis.

Primer name	Primer sequence (5′-3′)
NtUbiquitin-F	ATGAGTTGCGTGTTGCTCCT
NtUbiquitin-R	TACCTGTTGTACGGCCACTG
PqMYBF1-F	GGAAATGAATTGCTTCTGGA
PqMYBF1-R	AATATGAAATCGGCGAATGC
NtCHS-F	TGACACCCACTTGGATAGTTTAG
NtCHS-R	CGACCTCTGGAATTGGATCAG
NtCHI-F	GGATGATGGTGCGGTTGGTA
NtF3H-F	CCGACCTTACCCTTGGACTG
NtF3H-R	TCGAGTTCACCACTGCTTGA
NtF3′H-F	AGGCTCAACACTTCTCGT
NtF3′H-R	CATCAACTTTGGGCTTCT
NtFLS-F	GGTTAGGAGCCCATGAAATGA
NtFLS-R	CACAACACCAAGTGCCAAATC
NtDFR-F	AACCAACAGTCAGGGGAATG
NtDFR-R	TTGGACATCGACAGTTCCAG
NtANS-R	TGGCGTTGAAGCTCATACTG
NtANS-R	GGAATTAGGCACACACTTTGC

Table S3 The primers used for constructing vectors of subcellular localization assay.

Primer name	Primer sequence (5′-3′)
pCAMBIA2300-*PqMYBF1*-F	CGAGCTCGGTACCCGGGGATCCATGGGGAGGGCACCCTGTTG
pCAMBIA2300-*PqMYBF1*-R	CTTGCTCACCATGGTGTCGACAGAAAGAAGCCAAGCAACCA

Table S4 The primers used for constructing vectors of transcriptional activity test.

Primer name	Primer sequence (5′-3′)
pGBKT7-*PqMYBF1*-F	GGCCGAATTCCCGGGGATCCATGGGGAGGGCACCCTGTTGTG
pGBKT7- *PqMYBF1*-R	TGCGGCCGCTGCAGGTCGACTCAAGAAAGAAGCCAAGCAACC

Table S5 The primers used for constructing vectors of GUS staining analysis.

Primer name	Primer sequence (5′-3′)
PqFLS-SP1	CATGCGCGCGCGATCCTTGT
PqFLS-SP2	GGACAAAGAGTGCGTTTGGAAG
PqFLS-SP3	CAGTGTGAGGCTCAACTCCTAGT
PqFLS- pBI121-F	GCAACTAGTTACGCCAAGCTTTTGGAAGAGAACTATGATGC
PqFLS- pBI121-R	GGACTGACCACCCGGGGATCCGCTCTGTTTTCTCTGTTTTTG
PqCHS-pBI121-F	GCAACTAGTTACGCCAAGCTTAGATCGAATATTTTGATGGC
PqCHS-pBI121-R	GGACTGACCACCCGGGGATCCTTTCGCCGGAGACCACTGC
PqF3H-pBI121-F	GCAACTAGTTACGCCAAGCTTGAGAACAAATCGTCCCACC
PqF3H-pBI121-R	GGACTGACCACCCGGGGATCCGGTATAAGGAATTCTTGCTG

Table S6 The primers used for constructing vectors of dual-luciferase reporter assay.

Primer name	Primer sequence (5′-3′)
PqMYBF1-pGreenⅡ 62-SK-F	CCCCCGGGCTGCAGGAATTCATGGGGAGGGCACCCTGTT
PqMYBF1-pGreenⅡ 62-SK-R	GATTTCAGCGAATTGGTACCTCAAGAAAGAAGCCAAGCA
pGreenⅡ 0800-*pCHS*-LUC-F	GGCCCCCCCTCGAGGTCGACAGATCGAATATTTTGATGGC
pGreenⅡ 0800-*pCHS*-LUC-R	GCTCTAGAACTAGTGGATCCTTTCGCCGGAGACCACTGC
pGreenⅡ 0800-*pF3H*-LUC-F	GGCCCCCCCTCGAGGTCGACGAGAACAAATCGTCCCACC
pGreenⅡ 0800-*pF3H*-LUC-R	GCTCTAGAACTAGTGGATCCGGTATAAGGAATTCTTGCTG
pGreenⅡ 0800-*pFLS*-LUC-F	GGCCCCCCCTCGAGGTCGACTTGGAAGAGAACTATGATG
pGreenⅡ 0800-*pFLS*-LUC-R	GCTCTAGAACTAGTGGATCCGCTCTGTTTTCTCTGTTTTTG
